# Local connectivity of the resting brain connectome in patients with low back-related leg pain: A multiscale frequency-related Kendall's coefficient of concordance and coherence-regional homogeneity study

**DOI:** 10.1016/j.nicl.2019.101661

**Published:** 2019-01-14

**Authors:** Fuqing Zhou, Lin Wu, Linghong Guo, Yong Zhang, Xianjun Zeng

**Affiliations:** a Department of Radiology, The First Affiliated Hospital, Nanchang University, Nanchang 330006, China; b Department of Pain Clinic, The First Affiliated Hospital, Nanchang University, Nanchang, Jiangxi Province 330006, China; c Neuroimaging Lab, Jiangxi Province Medical Imaging Research Institute, Nanchang 330006, China

**Keywords:** Regional homogeneity, Local connectivity, Resting-state functional magnetic resonance imaging, Low back-related leg pain

## Abstract

Increasing evidence has suggested that central plasticity plays a crucial role in the development and maintenance of (chronic) nonspecific low back pain. However, it is unclear how local or short-distance functional interactions contribute to persisting low back-related leg pain (LBLP) due to a specific condition (*i.e.*, lumbar disc herniation). In particular, the multiscale nature of local connectivity properties in various brain regions is still unclear. Here, we used voxelwise Kendall's coefficient of concordance (KCC) and coherence (Cohe) regional homogeneity (ReHo) in the typical (0.01–0.1 Hz) and five specific frequency (slow-6 to slow-2) bands to analyze individual whole-brain resting-state functional magnetic resonance imaging scans in 25 persistent LBLP patients (*duration: 36.7* *±* *9.6 months*) and 26 healthy control subjects. Between-group differences demonstrated significant alterations in the KCC- and Cohe- ReHo of the right cerebellum posterior lobe, brainstem, left medial prefrontal cortex and bilateral precuneus in LBLP patients in the typical and five specific frequency bands, respectively, along with interactions between disease status and the five specific frequency bands in several regions of the pain matrix and the default-mode network (*P* *<* *.01, Gaussian random field theory correction*). The altered ReHo in the five specific frequency bands was correlated with the duration of pain and two-point discrimination, which were assessed using partial correlational analysis. These results linked the course of disease to the local connectivity properties in specific frequency bands in persisting LBLP. In future studies exploring local connectome association in pain conditions, integrated frequency bands and analytical methods should be considered.

## Highlights

• This study demonstrated significant interactions between disease status and five specific frequency bands in several regions.
• The alterations in ReHo of five specific frequency bands were related to the duration of disease and 2-PD in LBLP patients.
• It is useful to select specific frequency or the method of analyses to improve the detection of LBLP-related brain activity.

## 1 Introduction

Low back pain is an extremely common disorder, estimated to affect millions of individuals each year (Zhang et al., 2009). Most patients is nonspecific low back pain, though some patients have nerve root compression due to lumbar disc herniation (so called low back-related leg pain (LBLP)) (Kongsted et al., 2012). However, simple mechanical problems, such as herniated nucleus pulposus and herniated disk, cannot adequately explain the clinical symptoms of LBLP patients or the possible pathogenesis of the pain. In previous clinical studies, LBLP was found to be associated with increased disability and pain and poorer quality of life and recovery than patients with nonspecific low back pain (Konstantinou et al., 2013; Stynes et al., 2016). Additionally, approximately one-quarter to one-third of these LBLP patients continued to have pain after surgery (Konstantinou et al., 2015). Therefore, understanding the role of neural plasticity or rewiring of the brain in LBLP patients will improve our knowledge of pain symptoms and of the mechanisms underlying LBLP-associated central modulation.

In previous neuroimaging studies of low back pain, white matter structural damage (Buckalew et al., 2010; Shi et al., 2015; Ung et al., 2014), cortical atrophy (Cauda et al., 2014; Fritz et al., 2016) or thinning (Dolman et al., 2014) and pain-induced disturbance of functional activation were observed in several specific cortical and subcortical areas, including the pain matrix (somatosensory cortices (S1/S2) (Kong et al., 2013), insula and prefrontal cortices (Baliki et al., 2006; Kobayashi et al., 2009)) and higher-order brain systems, such as the default-mode network (DMN) (Baliki et al., 2008; Tagliazucchi et al., 2010).

In more recent studies, resting-state functional magnetic resonance imaging (rs-fMRI) has become a state-of-the-art method for decoding the local and remote functional properties of physiological and pathological conditions, including autism, depression, schizophrenia, chronic pain and others (Biswal et al., 2010). Functional connectivity (FC) is one of the most widely used and reliable statistical methods for describing the relationships among spatially remote areas in the temporal domain, reflecting the neuronal intrinsic activity level in functional communication between regions (van den Heuvel and Hulshoff Pol, 2010). Local FC, particularly on the scale of millimeters, defined as FC at a local spatial scale (10–15 mm (Sepulcre et al., 2010)), measures the functional interactions or synchronizations, between neighboring voxels or vertices. Although no consensus exists on which protocol is optimal for local FC analysis, the regional homogeneity (ReHo) method is becoming increasingly recognized for its multiscale nature (in frequency), reproducibility, sensitivity and reliability for characterizing local functional and organization. ReHo's neurobiological meaning is related to information processing complexity, brain development and contributions to neuropsychiatric disorders (Jiang et al., 2014; Jiang and Zuo, 2015; Zuo et al., 2013).

In this study, we hypothesize that the local property of intrinsic brain activity is impaired when LBLP patients experience persistent pain and paresthesia (numbness). For this aim, two ReHo methods for rs-fMRI based on Kendall's coefficient of concordance (KCC) and coherence (Cohe), proposed by Zang et al. (2004) and Liu et al. (2010), respectively, were used to explore the local FC of LBLP patients. Furthermore, in consideration of the frequency property of neuronal oscillations, we also generated ReHo maps within different frequency subbands to extract the neurophysiological basis of the local blood oxygenation level-dependent (BOLD) activities in LBLP patients. In previous studies, the frequency-dependent properties of ReHo measures were found to possibly arise from the varied cytoarchitecture or synaptic types in these areas and were associated with input selection, binding cell assemblies, consolidation and the combination of information (Baliki et al., 2011; Buzsáki and Draguhn, 2004; Di et al., 2013; Zuo et al., 2013). For pain, a controversial finding is that the alterations in the relatively high-frequency bands may have important physiological meanings in chronic somatic pain (Malinen et al., 2010), visceral pain (Hong et al., 2013) and fibromyalgia (Garza-Villarreal et al., 2015).

In accordance with the abovementioned situation, we used KCC- and Cohe-ReHo to explore local features of the resting brain connectome across multiple frequencies in patients with LBLP. Moreover, associations between ReHo and clinical evaluations of pain intensity and tactile discrimination ability were assessed.

## 2 Material and methods

### 2.1 Subjects

Thirty right-handed LBLP patients and age-, gender- and education-matched healthy controls (HCs) were recruited from our hospital and from the local community from Oct. 2016 to Jun. 2017. The LBLP patients were included according to the following criteria: (1) age 35–65 years old and volunteered to enroll in the study; (2) clinically and radiologically diagnosed with clear evidence of discogenic compression on a lumbar CT and/or MRI (>1 ruptured annulus fibrosus with compressed soft tissue); (3) pain (visual analogue scale (VAS) score > 4) with radiating pain in the buttock(s) and lower limb(s), particularly under increased abdominal pressure (*e.g.*, sneezing, coughing); and (4) failure to respond to conservative treatment with medications within at least the month prior, *e.g.*, anti-inflammatory drugs (Motrin, Advil and Naproxen) and acetaminophen (*e.g.*, Tylenol) without opioids, exercise and physical therapy (Malik et al., 2013). The exclusion criteria were as follows: (1) spinal stenosis due to calcifications on the spinal protrusion, lateral recess stenosis, spinal stenosis, pyriformis syndrome or sciatica due to unexpected cause of disc herniation; (2) previous spinal cord or canal infection, trauma, surgery or any other spinal abnormality; (3) any central neurological disorder, such as a history of epilepsy, stroke, dementia, vascular malformation or developmental malformation; (4) cardiovascular, cerebrovascular, liver, kidney, hematopoietic diseases or any systemic diseases; (5) diagnosis of lumbar disc herniation without clinical symptoms; and (6) excessive head motion during scanning, defined as head motion larger than 2.0 mm translation or 2.0° rotation.

All subjects gave written informed consent before the rs-fMRI scan, and this case-control study was approved by the Medical Research Ethics Committee and the Institutional Review Board of The First Affiliated Hospital of Nanchang University. All of the research procedures were performed according to the ethical principles of the Declaration of Helsinki and the approved guidelines.

### 2.2 MRI scan acquisition

All MR images were acquired with a Trio 3.0-Tesla Siemens scanner with a standard 8-channel head coil (Trio, Siemens, Munich, Germany). Foam padding was used to minimize head motion and machine noise, and subjects were asked to keep their eyes closed and not to fall asleep (confirmed by the postscan Epworth Sleepiness Scale (ESS) questionnaire). A high- resolution 3D-T_1_-weighted magnetization-prepared rapid gradient-echo (MP-RAGE) sequence (repetition time (TR)/echo time (TE) = 1900 ms/2.26 ms, field of view (FOV) = 215 mm × 230 mm, matrix = 240 × 256, thickness/gap = 1.0/0 mm and 176 sagittal slices) and rs-fMRI (TR/TE = 2000/30 ms, matrix = 64 × 64, FOV = 210 × 210 mm, 30 interleaved axial slices, 4 mm thickness, interslice gap of 1.2 mm, and 240 volumes over 8 min) were acquired.

Additional conventional T_2_-weighted and T_2_-fluid-attenuated inversion recovery (FLAIR) sequences were acquired to screen all subjects for anatomical brain abnormalities. Sagittal and axial conventional T_1_-weighted, T_2_-weighted and T_2_-fat suppression sequences were acquired for the lumbar spine and discs from L1 to S3 for the diagnosis of LBLP.

### 2.3 Rs-fMRI data preprocessing

The main preprocessing steps of rs-fMRI included the following: the first 10 volumes were discarded for signal stabilization and subject adaptation; then, slice timing, spatial realignment, head motion correction, individual registration between high-resolution T1 and echo planar imaging (EPI) images, T1 segmentation with the Diffeomorphic Anatomical Registration Through Exponentiated Lie algebra (DARTEL) and spatial normalization to register rs-fMRI data sets to the Montreal Neurological Institute (MNI) space were performed, along with resampling to 3 × 3 × 3 mm^3^ cube voxels; and head motion estimation, >2.0 mm of the maximal translation or 2.0° of the maximal rotation, was excluded from the final analysis. Linear detrending and nuisance linear regression (including the white matter, the cerebrospinal fluid and head motion parameters base on Friston 24-parameter model (Friston et al., 1996)) were performed, and a temporal bandpass filter was applied to reduce the effects of head motion and nonneuronal BOLD fluctuations. This preprocessing was performed using a toolbox for Data Processing & Analysis of Brain Imaging (Yan and Zang, 2010) (http://rfmri.org/dpabi) based on statistical parametric mapping (SPM12, http://www.fil.ion.ucl.ac.uk/spm/software/spm12/), which was run on MATLAB 8.4.0 (MathWorks, Natick, MA, USA).

### 2.4 Temporal filtering and ReHo analysis

(1) Predefined settings were used to calculate the ReHo; to investigate local features of the resting brain connectome in patients with LBLP, the preprocessing of the rs-fMRI data divided the data into a typical frequency band (0.01–0.1 Hz) and five specific frequency bands: slow-6 (0–0.01 Hz), slow-5 (0.01–0.027 Hz), slow-4 (0.027–0.073 Hz), slow-3 (0.073–0.198 Hz) and slow-2 (0.198–0.25 Hz), according to the Buzsáki framework (Buzsáki and Draguhn, 2004).

(2) Individual KCC-ReHo mapping: KCC of the predefined frequency band time series of each voxel relative to nearest neighbors (26 in this study) was calculated within the whole brain using the Resting-State fMRI Data Analysis Toolkit plus V1.2 (RESTplus V1.2, http://restfmri.net/forum/RESTplusV1.2). The KCC-ReHo detailed mathematical formula was as follows (Zang et al., 2004):

(1) $$\mathrm{KCC}=\frac{\sum_{i=1}^{n}R_{i}^{2}-n{\left(\bar{R}\right)}^{2}}{\frac{1}{12}K^{2}\left(n^{3}-n\right)}=12\frac{\sum_{i=1}^{n}{\left(\bar{R_{i}}\right)}^{2}}{\left(x^{3}-n\right)}-3\frac{\left(n+1\right)}{\left(n-1\right)}$$

where *R*_*i*=1. , *n*_is the sum rank of the *i*th time point, *n* is the number of temporal observations in the time series,$\bar{\;R_{i}}$is the mean of the *R*_*i*_across all the K neighbors and all temporal observations and *K* is the number of time series within a measured cluster (here, K = 27). According to this equation, a larger value of a given voxel (node) indicated higher local FC or network centrality in three aspects with the following advantages: acquired the connectivity strength interaction with nearest neighboring nodes; high-efficiency rank-based computation; and robust against noise by integrating the spatial domain (the mean-rank filter) and noise-filtering of the temporal domain (the order-rank filter) (Jiang and Zuo, 2015).

(3) Individual Cohe-ReHo mapping: calculating the Cohe-ReHo of the predefined frequency band time series of each voxel included the three following steps (Liu et al., 2010). First, power spectrum and cross-spectrum estimations were performed for any two time series in a given cluster using Welch's-modified periodogram averaging methods ((2), (3)):

(2) $$f_{\mathrm{xy}}^{\wedge \left(T\right)}\left(\lambda \right)=\frac{1}{N}\sum_{n=1}^{N}X_{n}^{\left(T\right)}\left(\lambda \right).Y_{n}^{⋆\left(T\right)}\left(\lambda \right)$$

(3) $$f_{x}^{\wedge \left(T\right)}\left(\lambda \right)=\frac{1}{N}{\left|X_{n}^{\left(T\right)}\left(\lambda \right)\right|}^{2}$$

where *X*_*n*_^(*T*)^(*λ*) is the discrete Fourier transform of the *n*_*th*_ segment of timeseries *x*(*t*). Second, estimation of coherence across predefined frequency bands and their band-averaged estimates of the cross spectrum and power spectra were calculated (in formula 4):

(4) $${\mathrm{Coh}}_{\mathrm{xy}}\left(\bar{\lambda }\right)=\frac{{\left|\sum_{\lambda }f_{\mathrm{xy}}\left(\bar{\lambda }\right)\right|}^{2}}{\sum_{\lambda }f_{x}\left(\bar{\lambda }\right).\sum_{\lambda }f_{y}\left(\bar{\lambda }\right)}$$

Finally, the averaged coherence coefficient of the cluster within the given cluster was calculated to represent the Cohe-ReHo of its center voxel (in formula 5, where *K*=27):

(5) $$\mathrm{Cohe}=\bar{\mathrm{Coh}}=\frac{2}{K\left(K-1\right)}\sum_{x=1}^{k-1}\sum_{y=x+1}^{K}{\mathrm{Coh}}_{\mathrm{xy}}\left(\bar{\lambda }\right)$$

Therefore, an individual Cohe-ReHo map was also generated for each subject using the RESTplus toolkit.

### 2.5 Clinical evaluation

Before the rs-fMRI scan, each subject agreed to the following clinical evaluations: the VAS (0−10) for pain intensity, the Japanese Orthopaedic Association (JOA) Back Pain Evaluation questionnaire (−6 to 29) to examine the impact of neuropathic or nociceptive pain on quality of life (Yonenobu et al., 2001), the Fugl-Meyer assessment for sensorimotor impairment measurement and the two-point tactile discrimination (2PD) test to assess tactile spatial resolution ability at the feet, and particularly at the hands (Boldt et al., 2014).

### 2.6 Statistical analysis

Before statistical analysis, the individual KCC-ReHo and Cohe-ReHo maps were generated *via Fisher's* r-to-z standardization within a whole-brain mask; then, the resulting data were further spatially smoothed using a 6-mm isotropic full width at half maximum (FWHM) Gaussian kernel.

Group mean z-values of KCC-ReHo or Cohe-ReHo were calculated for estimating the spatial patterns or distribution of the predefined frequency bands in the patients with LBLP and HC. To explore the differences in the KCC-ReHo or Cohe-ReHo between the LBLP and HC groups in the typical frequency band and five specific frequency bands, second-level random-effects two-sample *t*-tests were performed on individual zReHo data with smoothing in a voxel-by-voxel manner (*two-tailed, P* *<* *.01, Gaussian random field (GRF) theory correction with cluster-level P* *<* *.05*). To calculate the interactions between pain status and the five specific frequency bands on the ReHo map, we performed an ANOVA (flexible factorial design, 2 × 5) using SPM12 with the groups (LBLP and HC) as the between-subject factor and the frequency band (slow-2 to slow-6) as the within-subjects factor (*two-tailed, voxel-level P* *<* *.01, GRF correction with cluster-level P* *<* *.05*). All significant clusters were reported on the MNI coordinates, and T-values of the peak voxel were determined. Cohen's *d* was computed using a between-groups t-test value for effect size analysis. Cohen's *d* can be interpreted in terms of the percent of nonoverlap between two groups, while “d = 0.2,” “d = 0.5,” “d = 0.8” and “d = 2.0” indicated nonoverlaps of 14.7%, 33.3%, 47.4% and 81.1% in the two distributions, respectively.

Clinical characteristics and index statistics were analyzed using two-sample *t*-tests and *chi- square* tests in SPSS (release 13.0, SPSS Inc., Chicago, IL, USA) to explore differences between groups.

In addition, clinical associations were analyzed by partial correlational analyses in the SPSS 13.0 software between the clinical evaluation and zReHo values for brain areas that exhibited significant differences between the LBLP and HC groups, with the effects of age, gender and mean head motion as covariates (*P* < .05 with Bonferroni corrections).

## 3 Results

### 3.1 Clinical characteristics and indices

Among the participants, 3 patients and 4 healthy subjects were excluded due to excessive head motion. In addition, 2 patients were excluded for a vascular malformation and an infarction. Ultimately, a total of 25 persistent LBLP patients and 26 HC (age: LBLP: 55.16 ± 9.16 *vs.* HC: 53.38 ± 8.34 years, *P* = .473) subjects were selected for the group ReHo comparison. Among these patients, 25 (100%) patients presented with low back pain (*duration: 36.72* *±* *9.63 months*), 23 (92%) patients also presented with pain and numbness in a unilateral lower limb and 2 (8%) patients also presented with pain and numbness in bilateral lower limbs. Among the patients, the significantly low JOA Back Pain Evaluation scores (LBLP: *13.72* *±* *1.13 vs. HC: 28.96* *±* *0.04, P* *<* *.0001*) indicated an effect on quality of life due to neuropathic or nociceptive pain, and high VAS scores (LBLP: *5.78* *±* *0.21 vs. HC:0* *±* *0, P* *<* *.0001*) indicated tolerable or moderate pain. The mean Fugl-Meyer score was 19.2 ± 4.3 (range from 15.0 to 24.0). In the tactile spatial resolution ability assessment, decreased 2PD test scores in LBLP patients were observed in the right (30.60 ± 1.62 mm) and left (30.00 ± 1.17 mm) feet and the right (24.96 ± 1.22 mm) and left (26.24 ± 1.27 mm) hands, which indicated cortical reorganization of, for instance, somatosensory cortices. Finally, there were no significant differences in age (LBLP: *55.16* *±* *1.83 vs. HC: 53.54* *±* *1.66 years; P* *=* *.727*), gender (M/F: LBLP: *13/12 vs. HC: 14/12, χ*^*2*^ *test, P* *=* *.89*) or head motion (LBLP: *0.042* *±* *0.021* mm *vs. HC: 0.037* *±* *0.019* mm*; P* *=* *.467*) between the patients and HCs.

### 3.2 Spatial distribution pattern of ReHo in LBLP patients and healthy subjects

**Fig. 1:**
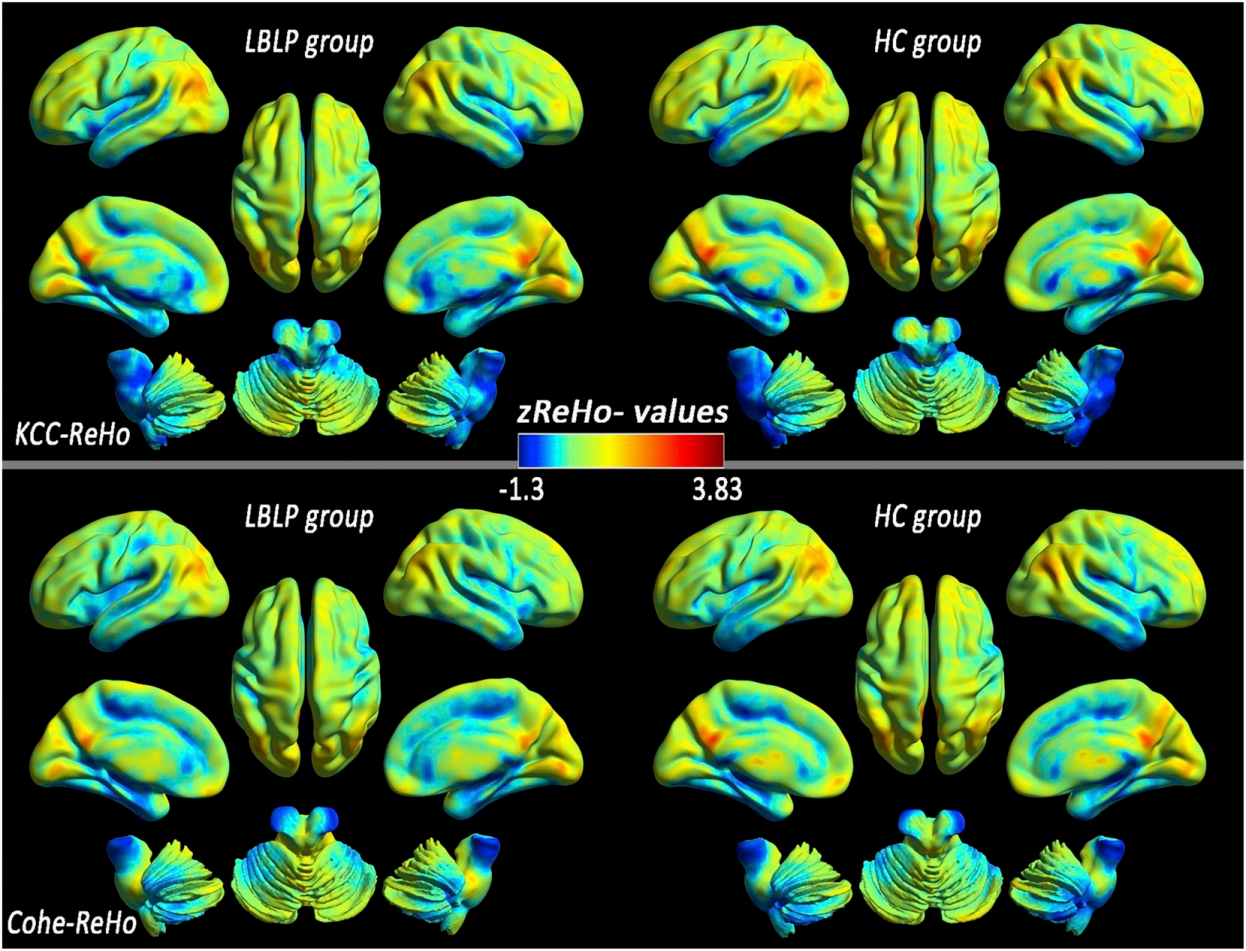
Similar distribution patterns of KCC-ReHo (top row) and Cohe-ReHo (bottom row) were observed at the group level for LBLP patients (left column) and healthy subjects (right column) in the typical frequency band (0.01–0.1 Hz).

**Fig. 2:**
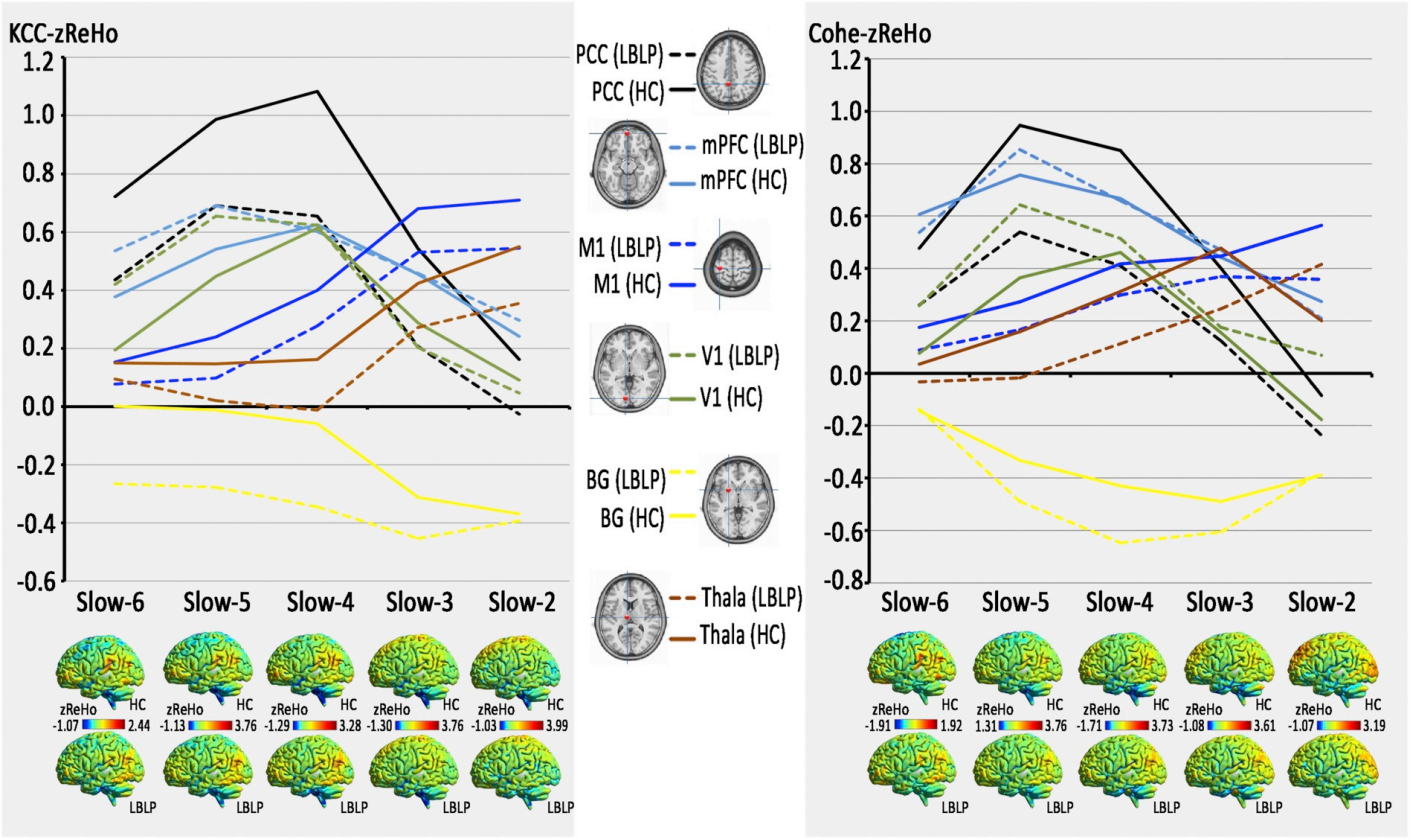
Distribution patterns and group differences (LBLP *vs.* HC) in several regions in terms of KCC-ReHo (left column) similar to Cohe-ReHo (right column), from slow-6 to slow-2. The line charts show dynamic changes from slow-6 to slow-2 in the representative nodes in the brain in the KCC-ReHo and Cohe-ReHo maps.

**Fig. A1:**
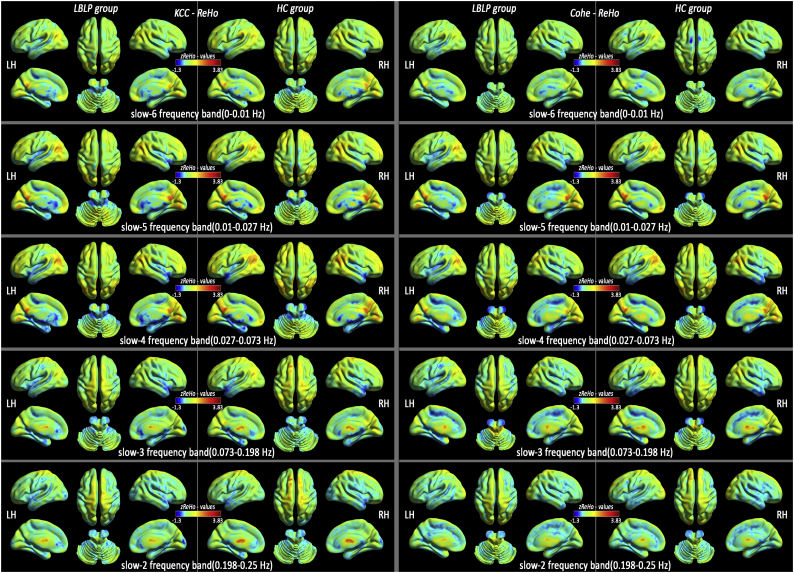
Similar distribution patterns of KCC-ReHo (left column) and Cohe-ReHo (right column) were observed at the group level among LBLP patients and healthy subjects in the five specific frequency bands: slow-6 (0–0.01 Hz), slow-5 (0.01–0.027 Hz), slow-4 (0.027–0.073 Hz), slow-3 (0.073–0.198 Hz) and slow-2 (0.198–0.25 Hz).

At the group mean level of the typical frequency band (0.01–0.1 Hz), the patients with LBLP were observed to have a roughly similar spatial distribution of KCC-ReHo or Cohe-ReHo to that of the HCs and higher KCC-ReHo and Cohe-ReHo in the regions (including the PCC/precuneus (PCUN), medial prefrontal cortex (mPFC) and bilateral inferior parietal lobule (IPL)) belonging to the DMN, indicating higher local connectedness (Fig. 1). In the five specific frequency bands, similar spatial distributions of KCC-ReHo or Cohe-ReHo were also observed between the LBLP and HC groups (Fig. A1), but the ReHo values of the slow-4 and slow-5 bands were higher than those of the other frequency bands (shown in Fig. 2).

### 3.3 Disease-related differences in ReHo within the typical and five specific frequency bands

**Fig. 3:**
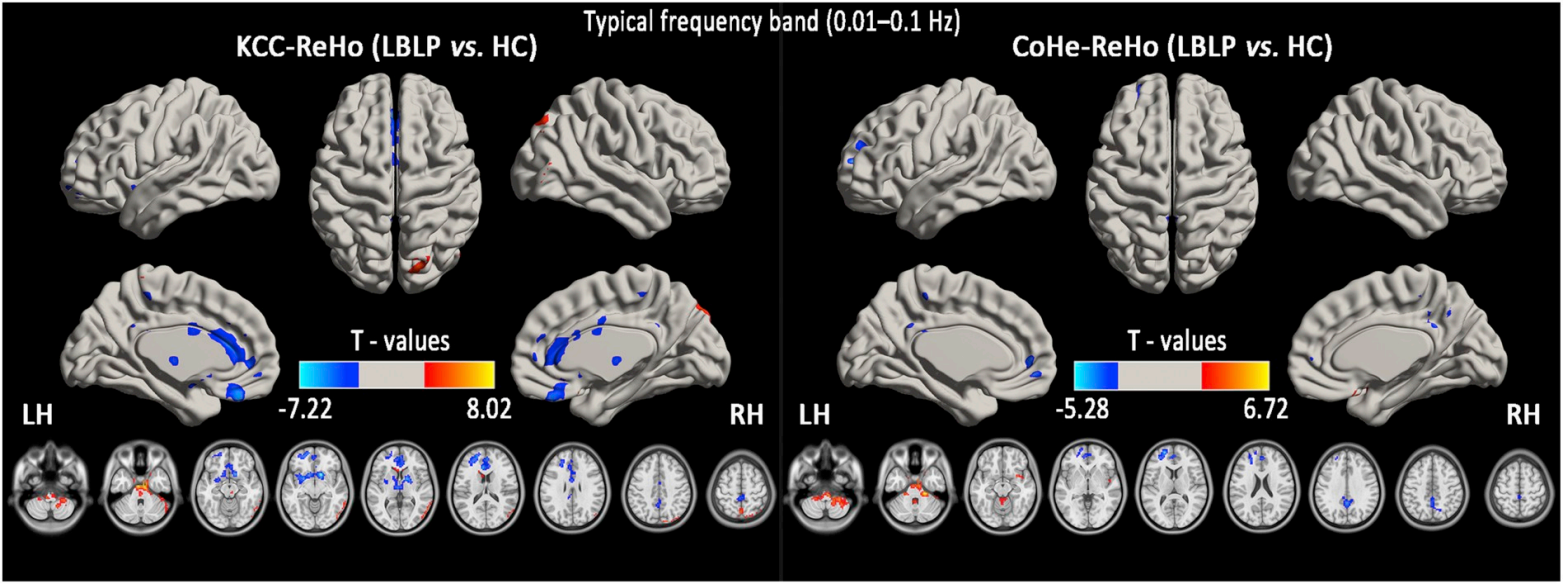
Group comparisons of the KCC-ReHo (left column) and Cohe-ReHo (right column) in the typical frequency band (0.01–0.1 Hz) between the LBLP patients and HCs *(two-tailed, voxel-level P* *<* *.01, GRF correction, cluster-level P* *<* *.05)*.

**Fig. A2:**
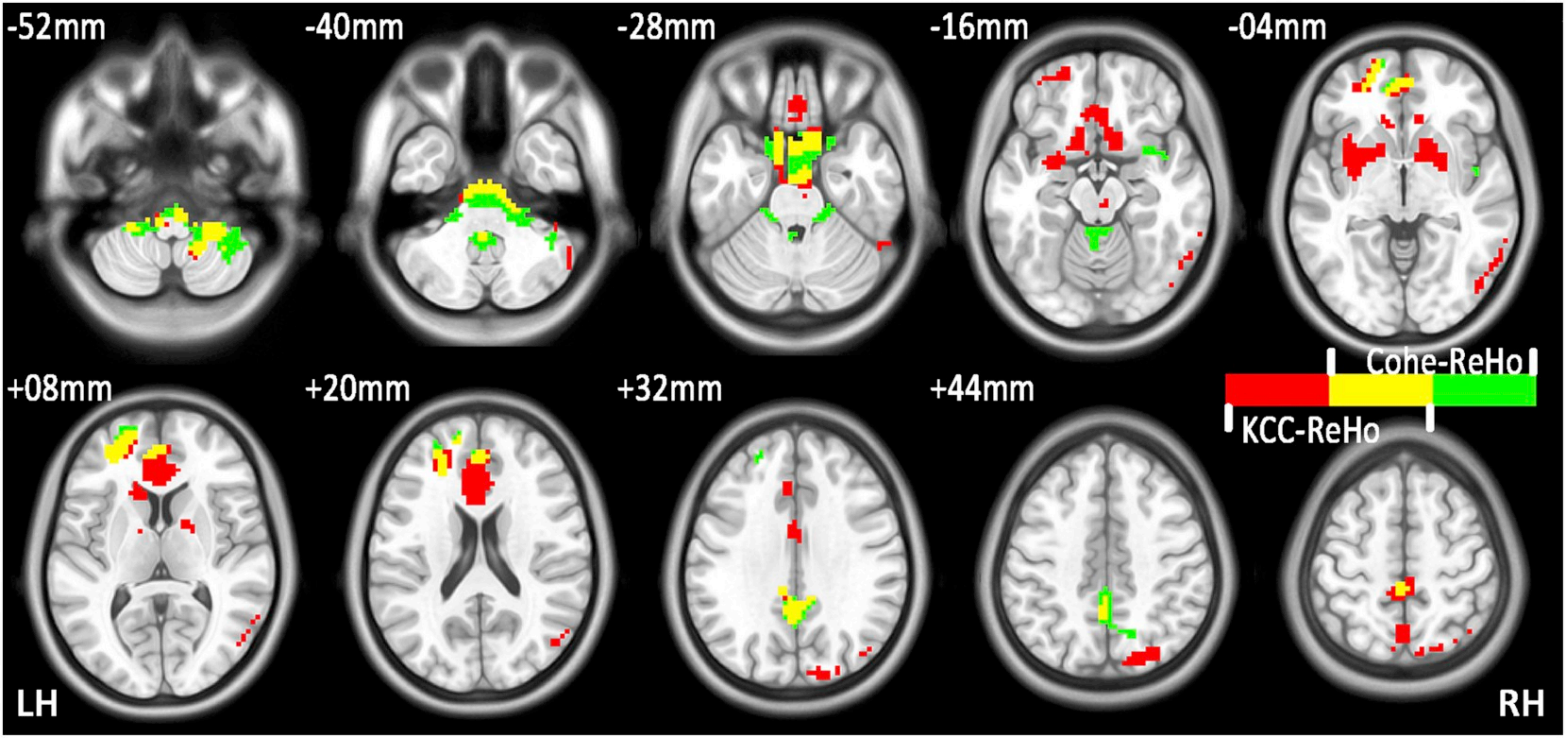
Disease-related alterations in the patients with LBLP observed in the KCC-ReHo and Cohe-ReHo analyses. *Red color indicates significant alterations in KCC-ReHo, green color indicates significant alterations in Cohe-ReHo and yellow color indicates the regions of both KCC- and Cohe-ReHo.*

Fig. 3 and Table 1 show the alterations in the spatial patterns of KCC-ReHo and Cohe-ReHo in the typical frequency band (0.01–0.1 Hz) using voxel-based analyses of the LBLP patients and HCs (*P* *<* *.01, GRF correction*). In the KCC- and Cohe-ReHo analyses of the typical frequency band, identical commonly altered regions were mainly found in the right cerebellum posterior lobe (CPL), brainstem, left mPFC and bilateral PCUN (shown in Fig. A2).

**Table 1:** Significant disease-related differences in the KCC-ReHo or Cohe-ReHo of the typical frequency band (0.01–0.1 Hz) between the LBLP patients and HCs (*two-tailed, voxel-level P* *<* *.01, GRF correction, cluster-level P* *<* *.05*).

Brain regions	BA	Peak T-scores	MNI coordinates			Cluster size (voxels)	Effect size (Cohen's *d*)
			x	y	z		
Altered KCC-ReHo in the typical frequency band (0.01–0.1 Hz) (LBLP *vs.* HC)							
Right cerebellum posterior lobe (CPL)		5.605	6	−36	−60	234	2.236
Brainstem/Midbrain		8.025	−6	−12	−36	408	2.732
Right precuneus/temporal-occipital joint	7,19,37	5.350	57	−63	−18	494	2.738
Bilateral basal ganglia		−7.221	12	12	−12	700	2.917
Left medial prefrontal cortex (MPFC)	10,11	−5.888	−21	57	9	382	2.273
Bilateral anterior cingulate cortex (ACC)	32,24	−5.118	−6	30	15	631	1.941
Bilateral precuneus (PCUN)	31,5,7	−4.327	0	−51	33	185	1.689

Altered Cohe-ReHo in the typical frequency band (0.01–0.1 Hz) (LBLP *vs.* HC)							
Right CPL/brainstem		6.717	21	−30	−33	1265	2.348
Left MPFC	10,32	−5.277	−21	57	9	458	2.030
Bilateral PCUN	31,7,5	−4.747	0	−48	33	282	1.852

**Fig. 4:**
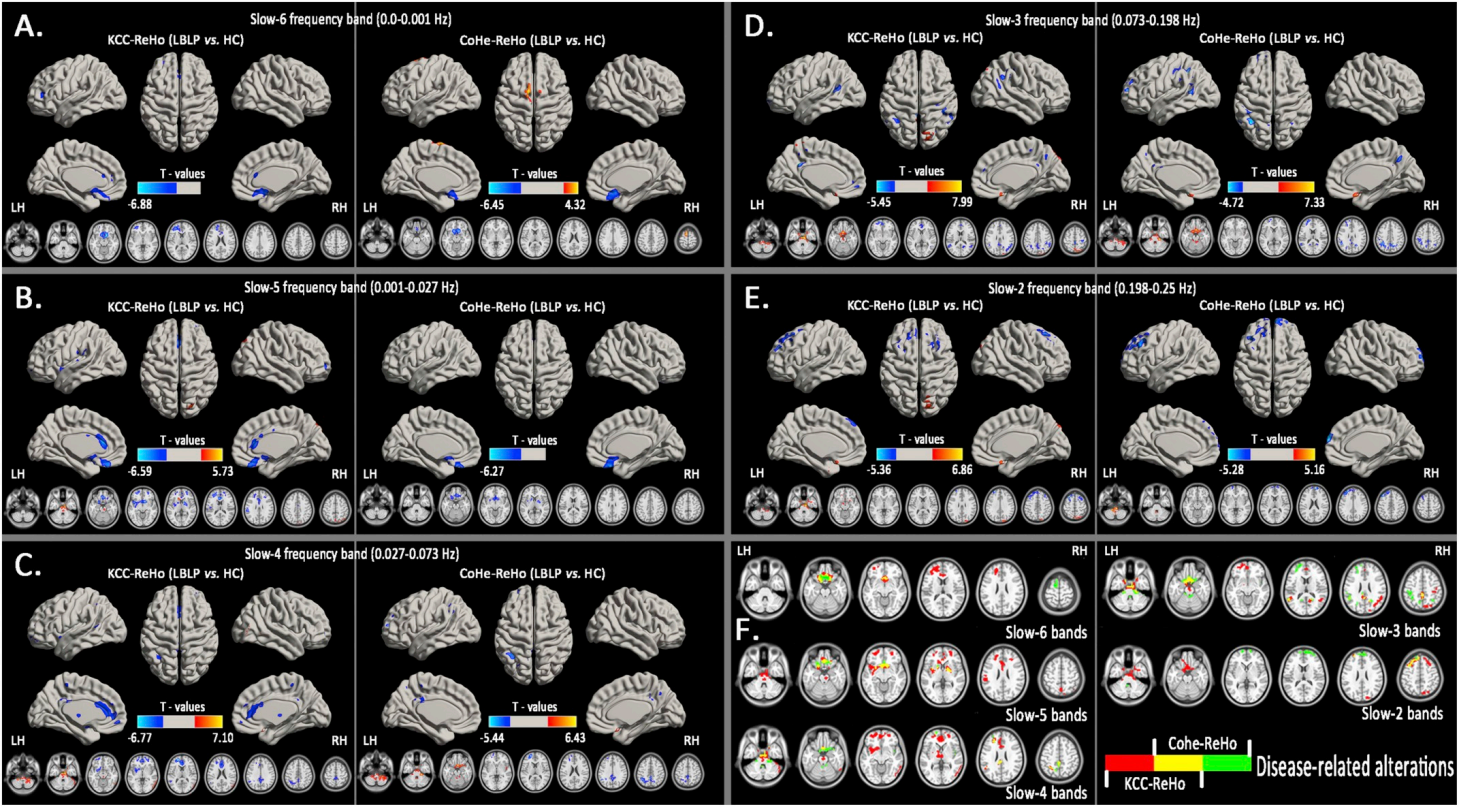
Group comparisons of the ReHo between the LBLP patients and HCs in the five specific frequency bands *(two-tailed, voxel-level P* *<* *.01, GRF correction, cluster-level P* *<* *.05)*. Note: A-E show alterations in the spatial patterns of KCC-ReHo (left column) and Cohe-ReHo (right column) in the five specific frequency bands: slow-6 (0–0.01 Hz), slow-5 (0.01–0.027 Hz), slow-4 (0.027–0.073 Hz), slow-3 (0.073–0.198 Hz) and slow-2 (0.198–0.25 Hz). F shows the disease-related alterations in the spatial patterns of KCC-ReHo (red color), Cohe-ReHo (green color) and the regions of both KCC- and Cohe-ReHo (yellow color).

Similarly, Fig. 4 show the alterations in the spatial patterns of KCC-ReHo and Cohe-ReHo in the five specific frequency bands using voxel-based analyses in the LBLP patients and HCs (*P* *<* *.01, GRF correction*). KCC-ReHo detected more regions (See Table 2) than Cohe-ReHo (See Table 3) in the relatively lower frequency bands, but in the higher frequency bands, identical commonly altered regions were observed mainly in the brainstem and bilateral gyrus rectus (Rec).

**Table 2:** Significant alterations of the KCC-ReHo of five specific frequency bands between the LBLP patients and HCs (two-tailed, voxel-level *P* < .01, GRF correction, cluster-level *P* < .05).

Brain regions	BA	Peak T-scores	MNI coordinates			Cluster size (voxels)	Effect size (Cohen's *d*)
			x	y	z		
Altered ReHo at slow-6 (0–0.01 Hz) band (LBLP *vs.* HC)							
Right rectus (REC)	25,12	−6.878	12	15	−15	453	1.978
Right medial prefrontal cortex (MPFC)/bilateral anterior cingulate cortex (ACC)	9	−4.795	−30	45	9	612	2.660

Altered ReHo at slow-5 (0.01–0.027 Hz) band (LBLP *vs.* HC)							
Brainstem		5.727	−6	−12	−36	213	1.846
Bilateral rectus/basal ganglia (BG)		−6.887	12	12	−12	996	3.726
Left MPFC	10,9	−5.203	−21	45	18	327	2.242
Right MPFC	10,9	−4.943	24	57	−3	208	2.023
Bilateral ACC	24,32,10	−5.761	−6	27	15	365	1.896
Bilateral precuneus	7,19	5.513	0	−66	60	209	2.007

Altered ReHo at slow-4 (0.027–0.073 Hz) band (LBLP *vs.* HC)							
Right cerebellum posterior lobe and brainstem		5.518	3	−33	−57	322	2.279
Brainstem		7.098	0	12	−21	434	2.697
Right temporal–occipital junction (TOJ)	37,21,19	5.122	57	−63	−18	201	1.903
Bilateral BG/thalami		−5.508	−12	12	−9	304	2.107
Bilateral ACC/left MPFC	32,10,24	−6.765	−18	54	9	1081	2.311
Left inferior parietal lobule (IPL)	40	−4.328	−51	−51	36	189	1.978
Bilateral precuneus	7,31	−4.310	−3	−51	36	246	2.273

Altered ReHo at slow-3 (0.073–0.167 Hz) band (LBLP *vs.* HC)							
Right cerebellum posterior lobe and brainstem		7.992	9	−12	−33	919	3.793
Bilateral ACC/MPFC	10,32,11	−5.069	−15	63	−6	263	2.008
Right IPL	40,39	−4.569	51	−48	21	440	2.341
Bilateral precuneus	7,31,5	−5.447	−3	−48	33	386	2.051
Bilateral superior parietal lobule	**7,19**	6.145	15	−81	51	293	2.455
Left TPJ	40,22	−4.839	−54	−51	12	205	2.226

Altered ReHo at slow-2 (0.167–0.25 Hz) band (LBLP *vs.* HC)							
Right cerebellum posterior lobe and brainstem		6.863	0	12	−21	752	4.274
Right precuneus/superior parietal lobule	10,7	4.516	18	−81	−48	232	2.377
Bilateral MPFC	8,6,9	−5.357	−30	27	57	553	2.570

Note: *ReHo* *=* *Regional Homogeneity; BA* *=* *Brodmann area; LBLP* *=* *low back-related leg pain; MNI* *=* *Montreal Neurological Institute (same as all figure and table).*

**Table 3:** Significant alteration of the Cohe-ReHo of five specific frequency bands between the LBLP patients and HCs (two-tailed, voxel-level *P* < .01, GRF correction, cluster-level *P* < .05).

Brain regions	BA	Peak T-scores	MNI coordinates			Cluster size (voxels)	Effect size (Cohen's *d*)
			x	y	z		
Altered ReHo at slow-6 (0–0.01 Hz) band (LBLP *vs.* HC)							
Right rectus (REC)	25,11,47	−6.445	15	15	−18	411	2.075
Bilateral superior frontal gyrus/supplementary motor area	6	4.324	−9	0	72	172	1.236

Altered ReHo at slow-5 (0.01–0.027 Hz) band (LBLP *vs.* HC)							
Bilateral rectus/basal ganglia (BG)		−6.272	0	27	−27	612	2.105

Altered ReHo at slow-4 (0.027–0.073 Hz) band (LBLP *vs.* HC)							
Right cerebellum posterior lobe and brainstem		6.431	3	12	−24	1209	2.013
Left MPFC	10	−5.444	−21	57	12	254	1.720
Left inferior parietal lobule (IPL)	40	−5.272	−51	−51	36	270	1.920
Bilateral precuneus	7,5	−4.219	0	−45	45	293	2.320

Altered ReHo at slow-3 (0.073–0.167 Hz) band (LBLP *vs.* HC)							
Right cerebellum posterior lobe and brainstem		7.732	21	−30	−33	1222	2.328
Left MPFC	10	−4.530	−24	60	12	234	2.001
Left IPL	40,7	−4.718	−39	−57	45	1026	2.527

Altered ReHo at slow-2 (0.167–0.25 Hz) band (LBLP *vs.* HC)							
Right cerebellum posterior lobe and brainstem		5.162	−18	−39	−51	243	2.138
Baliteral MPFC	10,9	−5.277	−36	30	45	747	1.925

*Note: ReHo* *=* *Regional Homogeneity; BA* *=* *Brodmann area; LBLP* *=* *low back-related leg pain; MNI* *=* *Montreal Neurological Institute (Same as all figures and tables).*

### 3.4 Interactions between disease status and the five specific frequency bands

**Fig. 5:**
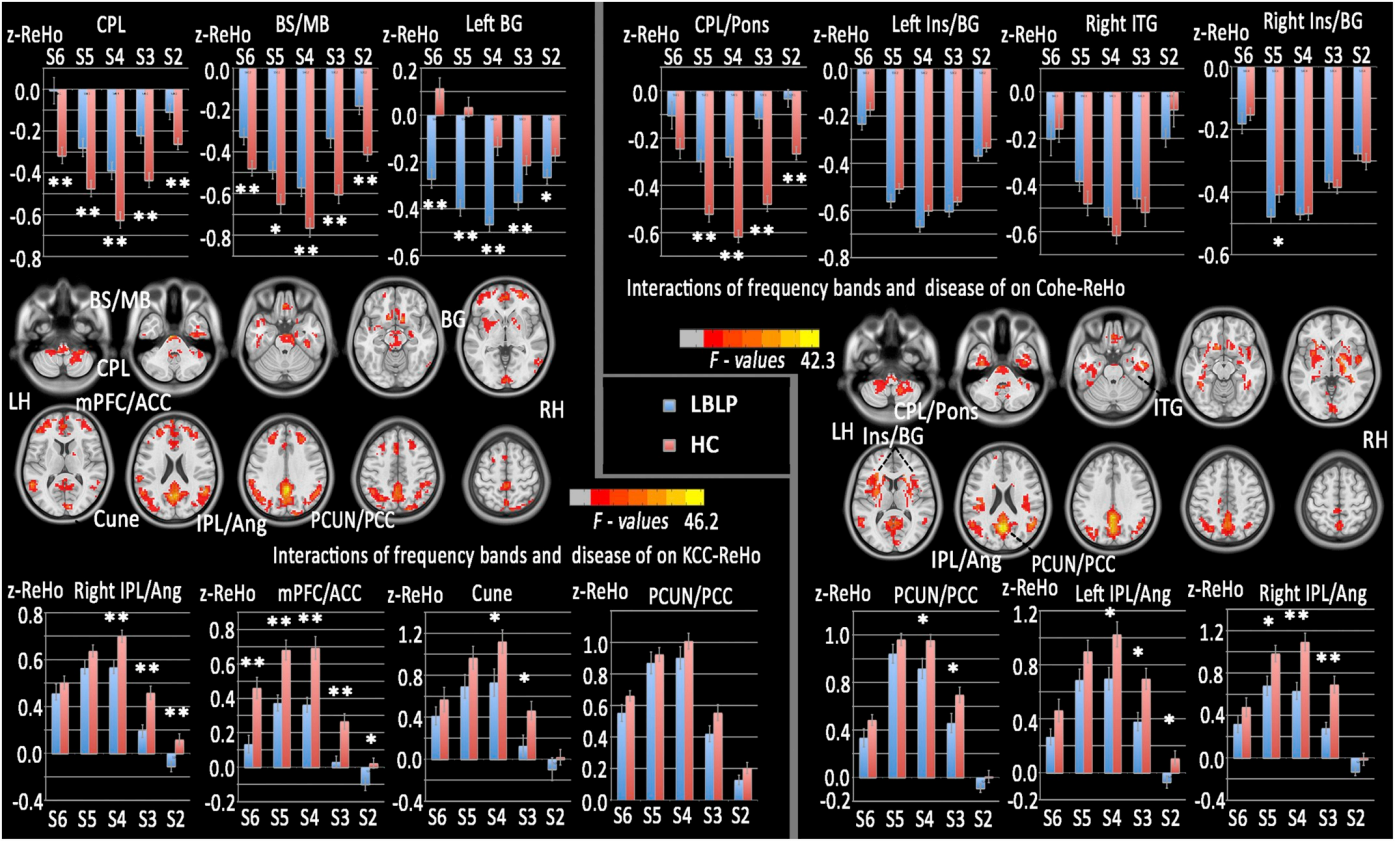
Interactions between the five specific frequency bands and disease status on ReHo. (A) Interactions between the specific frequency bands (slow-2 to slow-6) and group (LBLP patients and HCs) based on the ANOVA (flexible factorial design, 2 × 5, two-tailed, voxel-level *P* < .01, GRF correction, cluster-level *P* < .05). (B) The trend of AF in the rectal gyrus across the full-frequency band (0–0.25 Hz).

*ANOVA* analyses showed significant interactions between disease status and the five specific frequency bands in the right CPL, brainstem, basal ganglia (BG) and several regions of the DMN (including the mPFC, precuneus and IPL) in both the KCC- and Cohe-ReHo analyses (Fig. 5 and Table 4).

**Table 4:** Significant interaction between disease status and the five specific frequency bands on ReHo (flexible factorial design, 2 × 5).

Brain regions	BA	Peak t-scores	MNI coordinates			Cluster size (voxels)
			x	y	z	
Association between the frequency band (slow-2 to slow-6) and disease on KCC-ReHo						
Right CPL		28.262^※^	3	−36	−60	650
Brainstem/midbrain		42.788	9	−15	−33	489
Left BG		29.448	12	14	−12	611
Right IPL/angular cortex	40,39,19	29.801	54	−51	21	729
Bilateral mPFC/ACC	10,32	23.818	−30	48	12	1711
Bilatral cuneus	17,18	20.795	9	−90	6	176
Bilatertal precuneus/PCC	7,31,40	46.157	0	−48	33	2576

Association between the frequency band (slow-2 to slow-6) and disease on KCC-ReHo						
Right CPL		26.177	15	−42	−42	685
Left insula/BG	20	31.569	−15	21	15	1460
Right inferior temporal gyrus	20,21	25.218	51	−12	−27	303
Right insula/BG	13	33.908	21	21	6	1083
Bilateral precuneus/PCC	7,31,40	42.280	0	−63	21	1815
Left IPL/angular cortex	39,19	21.651	−57	−51	12	529
Right IPL/angular cortex	39,40,19	26.486	51	−57	21	375

*Note:*^※^*The F-test was statistically significant for an interaction between the ReHos of the five specific frequency bands (slow-2 to slow-6) and disease status. The T-test was statistically significant for particular analyses of interaction. All clusters were analyzed using a two-tailed test with a voxel-level threshold of P* *<* *.01, GRF correction, and cluster-level of P* *<* *.05.*

### 3.5 Correlations between abnormal ReHo values and clinical assessments

**Fig. 6:**
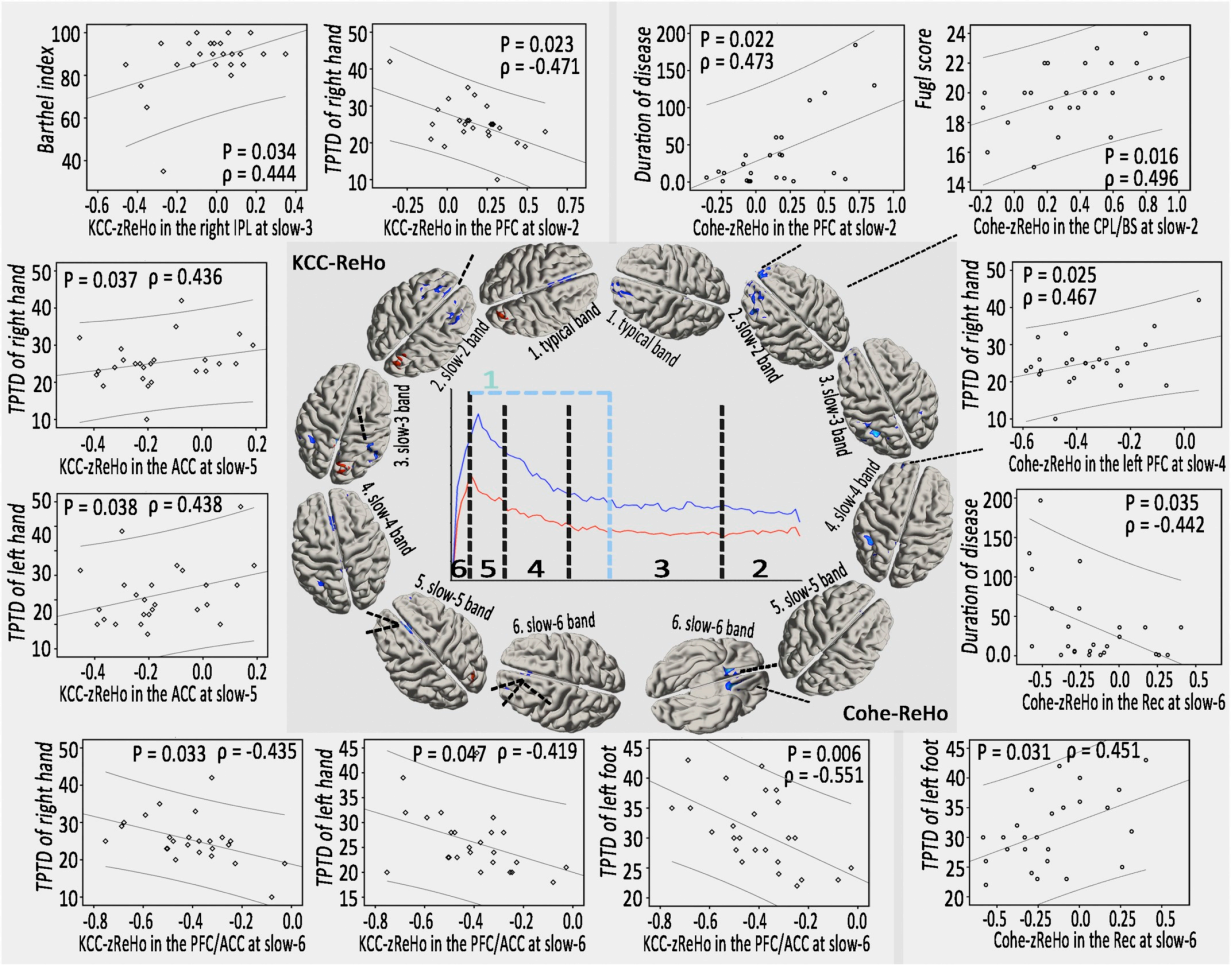
Associations between altered ReHo and clinical assessment scores in the LBLP patients (*P* < .05 with Bonferroni corrections).

Within the LBLP group (Fig. 6), the Cohe-zReHo of the bilateral prefrontal cortex (PFC) at slow-2 (*P* = .022) and the Cohe-zReHo of the bilateral Rec at slow-6 (*P* = .035) significantly correlated with duration of disease; the Cohe-zReHo in the CPL/brainstem (CPL/BS) at slow-2 significantly correlated with Fugl-Meyer scores (*P* = .016); and the Cohe-zReHo of the left PFC at slow-4 (*P* = .025) and the Cohe-zReHo of the bilateral Rec at slow-6 (*P* = .031) significantly correlated with 2PD values of the left foot. However, in the altered regions of KCC-ReHo, more alterations, similar to those in the bilateral PFC at slow-2 (*P* = .023), in the bilateral anterior cingulate cortex (ACC) at slow-5 (*P* = .037) and in the bilateral PFC/ACC at slow-6 (*P* = .033) significantly correlated with the 2PD of the right hand; values in the bilateral ACC at slow-5 (*P* = .038) and in the bilateral PFC/ACC at slow-6 (*P* = .047) significantly correlated with the 2PD of left hand; and values in the bilateral PFC/ACC at slow-6 correlated with the 2PD of the left foot (*P* = .006) (Fig. 6). No correlations were found between any of the clinical indices (duration of disease, JOA, VAS, 2PD, *etc.*) and altered KCC- or Cohe-ReHo in the typical frequency bands.

## 4 Discussion

In this study, we first investigated the local FC property in the typical and then five specific frequency bands using KCC- and Cohe-ReHo analyses; we then further demonstrated significant interactions between disease status and the five specific frequency bands in the right CPL, brainstem, BG and several regions of the DMN (including the mPFC, precuneus and IPL). Moreover, there were pain-related alterations in ReHo in the five specific frequency bands related to disease duration and 2PD assessments. Together, these findings provided a full view of the local connectivity property observed in BOLD waves, which is potentially useful for selecting specific frequencies or the method of ReHo analysis to improve the detection of LBLP-related brain activity.

### 4.1 Alterations in the ReHo in LBLP patients in typical frequency bands

As one subgroup of LBP, leg pain due to nerve root involvement is usually considered an obstacle to recovery or a marker of severity, particularly when associated with evidence of positive neurological findings. Although the mechanisms of intrinsic activity underlying the local connection disruptions observed in patients with LBLP are uncertain, several candidates should be considered. According to one hypothesis, LBLP patients may have both nociceptive and neuropathic pain. In this study, we demonstrated decreased ReHo in the left mPFC and bilateral PCUN and increased ReHo in the right CPL/BS in LBLP patients in typical frequency band analyses of KCC- and Cohe-ReHo. The PCUN and mPFC form a hub (or core) node of the DMN, and increased ReHo in experimentally induced low back pain (Zhang et al., 2014) and disrupted connectivity of the DMN in chronic low back pain (Baliki et al., 2008; Tagliazucchi et al., 2010) have been reported, together highlighting the impact of enduring pain and the cognitive or behavioral impairments accompanying chronic pain (Baliki et al., 2011; Loggia et al., 2013; Tagliazucchi et al., 2010). Disruptions in the dynamics of the DMN have also been demonstrated in the execution of simple attention-demanding tasks (Baliki et al., 2008) and can be functionally reversed by acupuncture treatment (Li et al., 2014). Recent rs-fMRI studies observed disruptions in the DMN in other pain conditions, including migraines (Tessitore et al., 2013), fibromyalgia (Garza-Villarreal et al., 2015), chronic somatic pain (Malinen et al., 2010) and diabetic neuropathic pain (Cauda et al., 2009). Furthermore, both increases and decreases in GM were found in the precuneus and dorsolateral prefrontal cortex (DLPFC, part of the mPFC) (Fritz et al., 2016; Luchtmann et al., 2014). These results provided evidence of intrinsic FC as a local property, and we reasoned that local connectivity changes at baseline might predispose patients with a lumbar disc herniation to develop persistent LBLP after the herniation and discogenic compression have resolved.

The brainstem and midbrain include the periaqueductal gray (PAG) and parabrachial nucleus (PB), which receive nociceptive input through the spinoreticular pathways (Dunckley et al., 2005). The CPL also receives information from the brainstem and plays an important role in fine motor coordination in the background of pain and paresthesia in LBLP patients (Diano et al., 2016). Increased ReHo in this area could indicate two different things, *i.e.*, either positive cooperativity in regional activity due to abnormal information inputs or negative “side effects”, followed by structural injury to inhibitory interneurons. Similarly, increased ReHo or hyperactivation has been observed in other pain disorders (Giesecke et al., 2004; Zhang et al., 2014). In this study, significant pain and paresthesia (numbness) were observed in the LBLP patients, implying that hyperactivity was involved in information processing for interpretation. Here, we argue that the former view best captures the essence of increased ReHo, a position that agrees well with the pain and paresthesia (numbness) in the patients.

### 4.2 Comparison of the alteration patterns between KCC- and Cohe-ReHo in LBLP patients

Local FC is defined by the temporal similarity or coherence of the BOLD time series within a set of a given voxel's nearest neighbors. Beyond other FC metrics, ReHo represents the most efficient, reliable and widely used index, KCC (Zang et al., 2004) and coherence-based (Liu et al., 2010) algorithms to measure the local synchronization of rs-fMRI signal. In the typical frequency bands, similar alterations in spatial patterns were observed in the CPL and brainstem/midbrain in KCC- and Cohe-ReHo analyses, but KCC-ReHo is more sensitive than Cohe-ReHo in detecting the differences in the BG and ACC. These preliminary results suggested that KCC-ReHo is superior to Cohe-ReHo in LBLP patients. Our study was different from previous studies, which detected differences between resting-state conditions [eyes open (EO) *vs.* eyes closed (EC)] (Liu et al., 2010) and detected abnormal local synchronization between two groups [attention deficit hyperactivity disorder (ADHD) patients *vs.* normal controls] (Zang et al., 2004). In the finding of the latter, Cohe-ReHo is more sensitive than KCC-ReHo due to KCC-ReHo being susceptible to random noise induced by the phase delay among the time courses (Liu et al., 2010). However, further investigations are still needed to elucidate the sensitivity and specificity of these methods in LBLP patients.

### 4.3 Interaction between frequency-based ReHo and disease changes in LBLP patients

In this study, significant interactions were identified between frequency-based ReHo in the five specific frequency bands and disease status in the pain matrix (the right CPL, brainstem/midbrain and left BG belong to the pain conducting system) and the DMN (mPFC/ACC, precuneus/PCC, cuneus and right IPL), suggesting that different frequency bands may have specific pathological relevance in this region. This notion was demonstrated in *post hoc* analyses of the region of interactions. In a previous study, the neuronal oscillations in the human brain proposed a spectrum of oscillatory bands for the implementation of functioning in human cognition. Zuo et al. (2013) and Song Song et al. (2014) found that ReHo in cortical areas was higher, and more frequency-dependent properties or increased richness in scales of ReHo measures across different scales of the frequency subbands were found. The distinct frequency-specific ReHo properties of different brain areas may arise from the various cytoarchitectural or synaptic types in these areas (Jiang and Zuo, 2015; Song et al., 2014; Zuo et al., 2013). Regarding the intrinsic activity, higher frequency fluctuations reflect local neural activity due to a lower magnitude of power, while lower frequency fluctuations reflect long-distance neural activity due to a higher magnitude of power (Buzsáki and Draguhn, 2004; Chang and Glover, 2010; Hipp and Siegel, 2015).

Therefore, in the *post hoc* ReHo analyses of the five specific frequency bands, the altered pattern in LBLP patients implied an interaction of pain (nociceptive and neuropathic) and paresthesia (numbness) in the frequency-related alterations in specific brain regions, including distributed changes in processing long-distance neural activity and local neural activity in the patients with LBLP. Baliki et al. (2011) found a graded shift in power from low- to high-frequency bands in the visual ventral stream, suggesting that a closed relationship exists between the anatomical structures and the full spectrum of intrinsic oscillations.

Our finding may underlie the neurophysiological basis of the local BOLD activities and the functional specificity of different brain regions. These findings suggest that specific frequency ranges, even high-frequency bands, should be selected to detect pain-related intrinsic activity in future studies of LBLP patients.

### 4.4 Correlations between clinical indices and abnormal ReHo in specific frequency bands

For LBLP patients, the KCC-ReHo in several brain regions, such as the bilateral ACC, at slow-5 correlated with the 2PD of the right and left hands, while the Cohe-zReHo of the left PFC at slow-4 correlated with the 2PD of the left foot. Together, these results suggest that decreased local connectivity is associated with decreased tactile spatial resolution ability. There were no correlations between the KCC-ReHo or Cohe-ReHo of the typical frequency bands with the clinical variables (duration of disease, JOA, VAS, 2PD, *etc.*) in LBLP patients, implying the lower clinical relevance of ReHo in the typical frequency bands.

In addition, the associations were observed between altered KCC-ReHo at slow-2, slow-3 or slow-6 and the 2PD of the hand or foot and between Cohe-ReHo at slow-2 or slow-6 and disease duration, suggesting that the altered ReHo of the relatively high-frequency bands also should not be ignored. For pain, previous studies found that the alterations in the relatively high-frequency bands may have important, though controversial, physiological meanings, including in chronic somatic pain (Malinen et al., 2010), visceral pain (Hong et al., 2013) and fibromyalgia (Garza-Villarreal et al., 2015). This study also supported this opinion.

### 4.5 Limitations

There were several limitations and methodological issues in this study. First, this was a pilot study with a relatively small sample that consisted of mostly middle-aged to elderly LBLP patients. In this susceptible population, individuals <65 years old were included in the ReHo analyses, which could have prevented interference from age-related factors, such as brain atrophy. Second, only KCC- and Cohe-ReHo, two 3D methods, were applied in this study. Another alternative method, 2D ReHo, has not been used to analyze the alteration in LBLP patients. The 2D ReHo method was developed by Zuo and others (Zuo et al., 2013) by extending the computation onto the cortical mantle. Third, the study's cross-sectional design might have an impact on determining cause-effect relationships.

## 5 Conclusion

In this study, we showed disease-related differences in the ReHo in the pain matrix and DMN within the typical and five specific frequency bands and interactions between disease status and the five specific frequency bands. Associations between the altered KCC- and Cohe- ReHo and the clinical assessment scores in the specific frequency bands are potentially useful for selecting specific frequencies or the method of ReHo analyses to improve the detection of LBLP-related brain activity.

## Author Contributions

FZ and YZ designed the study. LW, LG and YZ acquired the data. XZ and FZ processed the neuroimaging data. FZ performed the statistical analyses. All authors contributed to data interpretation and writing of the article.

## Conflict of interest

The authors declare no conflicts of interest with respect to the publication of this article.
